# Design of parallel cascade controller for nonlinear continuous stirred tank reactor

**DOI:** 10.1038/s41598-025-89455-6

**Published:** 2025-02-20

**Authors:** Mohammad Atif Siddiqui

**Affiliations:** Department of Electrical Engineering, Integral University, Lucknow, 226026 India

**Keywords:** Parallel cascade control structure, Series cascade control configuration, Nonlinear continuous stirred tank reactor, Engineering, Mathematics and computing

## Abstract

This work presents an approach to control the temperature of a nonlinear continuous stirred tank reactor (NCSTR) through parallel cascade control structure (PCCS). For the first time, PCCS is used to control the temperature of NCSTR by (1) modelling the dynamic behavior of CSTR with a recirculating jacket heat transfer system into a third order unstable transfer function and (2) using the model matching technique to synthesize the controller parameters. The controller of the secondary loop of PCCS is designed to achieve enhanced regulatory performance whereas, the primary loop controller is designed for better setpoint tracking. The closed loop performance of the proposed method is evaluated by carrying out simulation on the differential equation of the NCSTR and comparing it with other structures such as series cascade control structure (CCS) and parallel control structure (PCS). The response shows that the proposed method provides satisfactory performance in nominal, perturbed and noisy conditions.

## Introduction

A Nonlinear Continuous Stirred Tank Reactor (NCSTR) exhibits different dynamics such as linear, nonlinear, complex, etc. when operated in various functioning regions^1^. If NCSTR are persevered or handled at substantially higher transfiguration rates, the frugality and efficiency of the NSCTR are optimized. The similar achievement is also envisaged under the effect of load disturbances^2^. To achieve such a performance, proper controller designed technique^3–7^ in a suitable structure is essential. Many authors^8–10^ have proposed techniques to obtained the desired performance for NCSTR by considering the various dynamical models in a single loop configuration.

Researchers have controlled NCSTR by considering either one or two state model of NCSTR. The two-state model of NCSTR is regulated by Kumar and Sree^11^ and Lee et al.^12^ in a single feedback configuration. The controllers are designed using NCSTR model having double integrating dynamics with inverse response and internal model control (IMC) principle. Kumar and Sree^11^ showed improved performance over Lee et al.^12^ in terms of error minimization and overshoot. To further enhance the overall performance of the two-state model of NCSTR, Begum et al.^13^ and Novella-Rodriguez et al.^14^ have considered first and second order unstable model of NCSTR, respectively. The parameters of the controller are derived based on maximum sensitivity and model parameters. It is also to note that in the above-mentioned literature, the energy around the jacketed NCSTR that results into third state model have been overlooked.

The third state of the NCSTR have been considered by many researchers^8,10,15–17^ that is energy in the jacket around the NCSTR. Although the third state adds more dynamics to the model of NCSTR, it becomes further difficult to design the controllers and control it in a single loop configuration. A viable solution is to use cascade control structure (CCS) where the energy around the jacket is used as a feedback to enhance the disturbance rejection properties^10^. This benefits has been capitalized by Uma et al.^18^ where they have used Smith predictor based CCS with three controllers to control the three state NCSTR. The controllers are synthesized using IMC and direct synthesis scheme. Bhaskaran and Rao^3^ have controlled three state NCSTR using modified CCS with three controllers and three filters. The parameters of the controller and filters are obtained using predictive scheme, IMC scheme, direct synthesis scheme and unstable model of the NCSTR. In these works, jacket temperature is used as the manipulating variable and reactor temperature is the controlled variable. Choosing jacket temperature as the manipulating variable have few drawbacks such as slower response, complicates the control system and cost of the setup increases due to mixing valve or heat exchanger.

To overcome these issues, jacket makeup flowrate can be used as manipulated variable to control the reactor temperature of the three state NCSTR. Siddiqui et al.^2^, controlled NCSTR in CCS by using jacket makeup flowrate as manipulated variable, stable NCSTR model of order 3 and frequency response matching technique. Their technique showed superior performance over the method of Jeng and Lee^19^. Recently, Siddiqui et al.^20^ presented a comparative analysis of various structures such as single, series cascade, sliding mode and parallel control structures to control the NCSTR using jacket makeup flowrate as manipulated variable and reactor temperature as controlled variable. The model used in^20^ is a third order unstable process with inverse response dynamics as it captures better dynamics of NCSTR^21^. Among all these structures, the CCS is best suited to handle the NCSTR in a satisfactory way but at the cost of slower response to disturbance in secondary loop and limited adjustability in prioritizing control of the reactor temperature.

Another structure known as parallel cascade control structure (PCCS) has better load disturbance capabilities as compared to CCS because the disturbance and manipulated variable influence the secondary and primary responses, simultaneously. Unlike the CCS, the secondary loop plays an important part in contributing in load disturbance rejection performance, enhancing the dynamic performance of the PCCS, enhancing flexibility in control design as both loops are more independent, reducing risk of controller interaction and instability as the primary and secondary loops are decoupled, faster response to setpoint changes and reduced risk of saturation or nonlinearity in the control system.

Many researchers^22–24^ have used PCCS and controlled NCSTR by considering the model mentioned in^25^. Santosh and Chidambaram^22^ have used coefficient matching technique to design secondary loop P controller and primary loop PI controller of PCCS. Their method requires approximation of the actual process into first order systems thereby resulting in less robust performance. Further enhance performance is achieved by Raja and Ali^24^, where they have modified the PCCS structure and designed three controller based on IMC approach and unstable second order model parameters. Recently, Pashai and Bagheri^26^ have modelled NCSTR into a unstable SOPDT and used Smith Predictor based PCCS with fractional order controllers to control the concentration of NCSTR. Improved performance have been obtained as to compared to Raja and Ali^24^ and Padhan and Majhi^23^. In these literatures, none of the authors have controlled the temperature of NCSTR through PCCS by regulating the flow in the jacket around the NCSTR. Also, all simulations have been performed on the transfer function model of NCSTR, to be more realistic, it is desirable to perform simulation on non-linear differential equations of NCSTR.

Based on the above-mentioned works related to PCCS, the subsequent conclusions have been drawn:

1. Very limited literature is available for the control of NCSTR through parallel cascade control structure.
2. It should be noted that most of the works pertaining to controller design for NCSTR in PCCS are restricted to stable/unstable models having first or second order dynamics which in-fact, cannot depict the dynamics of NCSTR well enough. The reason being that most of the controller tuning approach are established either on stable/unstable models having first or second order dynamics.
3. These methods may be applied to control NCSTR model of third order unstable/stable dynamics by approximating the order of the model into lower and then designing the controller, however, this could lead to a decline in control performance.
4. Controller design approaches for controlling a three-state NCSTR model are typically customized to distinct process dynamics, such as first-order, second-order, or integrating dynamics with or without time delay. Only a few research is available that can control NCSTR process having various dynamics in a comprehensive and unified manner.
5. Complex structures of PCCS are used to control the NCSTR. But, in process industry, a simple structure with few controllers is usually preferred.
6. Closed loop performance of NCSTR through PCCS are inferior and requires additional improvement.

Hence, in this regard, a simple parallel cascade control structure is designed to control the temperature of the reactor by regulating the flow of liquid in the jacket around the NCSTR. The controllers of the PCCS are designed using the unstable third order model of NCSTR (which is the first implementation of PCCS for NCSTR with third order model dynamics) where the manipulated variable considered is jacket flowrate. Operating NCSTR in unstable region have several advantages related to prompt response to disturbances, capacity to optimized process efficiently, enhanced conversion rates and greater reaction rates. To achieve these advantages, the PI/PID controllers of the PCCS are designed by considering the unstable dynamics in the design procedure. The PI controller of the secondary loop is synthesized by choosing the desired closed loop model for load disturbance rejection (DCLM_FLD). The primary loop PID controller is schemed by considering the desired closed loop model for setpoint tracking ((DCLM_FST)) and placing the pole at desired position to achieve desired transient performance. The controllers are then approximated into PI/PID controller by applying the model matching technique in the frequency region to enhance the steady state performance. Finally, simulations on nonlinear differential equation of NCSTR are performed to show the effectiveness of the suggested approach in PCCS. The control of NCSTR is also carried out with other structures like CCS and parallel control structure (PCS). It is found that the PCCS performs superior in terms of load disturbance rejection and setpoint tracking of NCSTR as compared to CCS and PCS.

The noticeable attributes of the proposed work are:

(i) A maiden use of parallel cascade control structure to control the temperature of the NCSTR with makeup flowrate in jacket as manipulated variable is proposed.
ii) In PCCS, for the first time, the controllers are design considering the unstable NCSTR model with third order dynamics of three state NCSTR and using model matching technique in the frequency domain. The method is directly applicable to the system without approximating it to lower order system.
(iii) A detailed comparative analysis between PCCS, CCS and PCS has been conducted by carrying out simulation on nonlinear differential equations of NCSTR.

In “NCSTR dynamics and modelling”, the NCSTR dynamics and modelling are presented. The controller design approaches for PCCS are explained in “Controller design of PCCS” and “Robustness analysis” discusses the robustness analysis. The simulation study and conclusion is represented in “Simulation study” and “Conclusion”, respectively.

## NCSTR dynamics and modelling

**Fig. 1:**
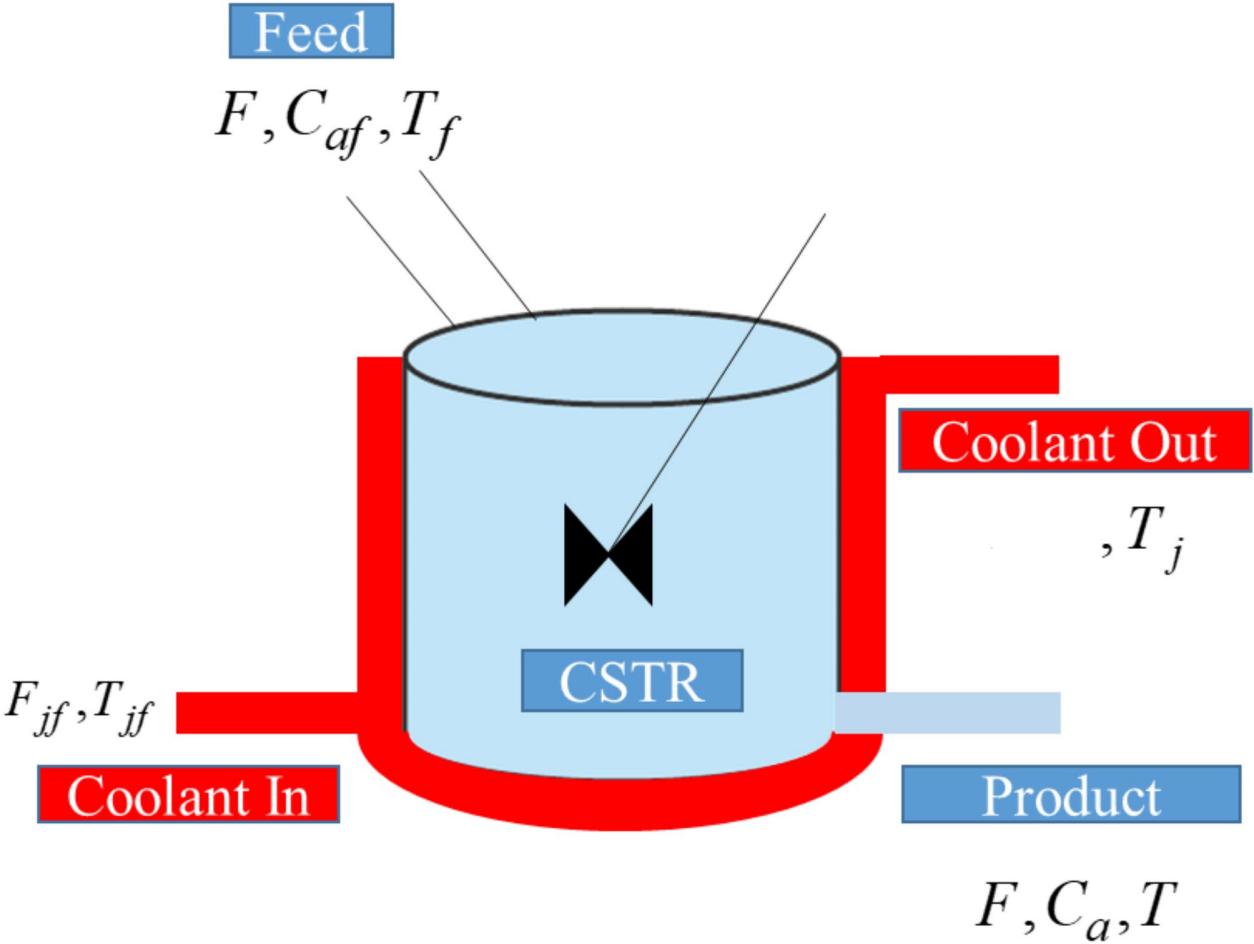
Jacketed NCSTR.

In this work, the control problem of NCSTR is considered and is shown in Fig. 1.

The basic purpose of NCSTR is to convert input feed into desirable product by permitting the stream of fluids uninterruptedly fed into the reactor and mixing them. In this process, the stream that comes out comprises of the fluid having constant concentration and temperature. The jacket is attached about the reactor where the fluid flows to regulate the temperature of the NCSTR. The reaction inside the NCSTR is represented in the form of differential equations as

1 $$\frac{{d{C_a}}}{{dt}}=\frac{F}{V}\left( {{C_{af}} - {C_a}} \right) - {k_0}\exp \left( {\frac{{ - {E_a}}}{{RT}}} \right){C_a},$$

and

2 $$\frac{{dT}}{{dt}}=\left( {{T_f} - T} \right)\frac{F}{V}+\left( {\frac{{{k_0} - \Delta H}}{{{C_\rho }}}} \right)\exp \left( {\frac{{ - {E_a}}}{{RT}}} \right)\frac{{{C_a}}}{\rho } - \frac{U}{{{C_p}}}\left( {T - {T_j}} \right)\frac{{{A_r}}}{{V\rho }},$$

where *A*_*r*_, *T*_*f* ,_*T*,* C*_*af*_, *U*,* Ca*,* ko*,* E*_*a*_, *F*,* C*_*p*_, *R*, $- \Delta H$,$\rho$and *V* are the area of heat transfer, feed temperature, reactor’s temperature, feed concentration, heat transfer coefficient, component’s concentration of A, frequency factor, activation energy, intake flow rate, specific heat capacity, heat of reaction, ideal gas constant, density and reactor’s volume, respectively. An additional energy around the cooling jacket is expressed as

3 $$\frac{{d{T_j}}}{{dt}}=\left( {{T_{jin}} - {T_j}} \right)\frac{{{F_j}}}{{{V_j}}}+\frac{U}{{{C_{pj}}}}\left( {T - {T_j}} \right)\frac{{{A_r}}}{{{V_j}{\rho _j}}},$$

where *T*_*j*_, *F*_*j*_ and *T*_*jin*_ represents the jacket temperature, flowrate through the jacket and the jacket inlet temperature, respectively.

By considering a static energy balance, a relationship is established between the jacket makeup flowrate (*F*_*jf*_) and the jacket inlet temperature as

4 $${T_{jin}}={T_j}+\left( {\frac{{\left( {{T_{jf}} - {T_j}} \right)}}{{{F_j}}}} \right){F_{jf}}.$$

Here, *T*_*jf*_ is jacket inlet temperature input. Putting the Eq. (4) into Eq. (3) yields

5 $$\frac{{d{T_j}}}{{dt}}=\left( {{T_{jf}} - {T_j}} \right)\frac{{{F_{jf}}}}{{{V_j}}}+\frac{U}{{{C_{pj}}}}\left( {T - {T_j}} \right)\frac{{{A_r}}}{{{V_j}{\rho _j}}}.$$

For additional comprehensive explanations of the NCSTR dynamics, kindly, refer to^20^.

For Eqs. (1, 2, 5), the following deviation variables represent the states (x)$$x=\left[ {\begin{array}{*{20}{c}} {{x_1}} \\ {{x_2}} \\ {{x_3}} \end{array}} \right]=\left[ {\begin{array}{*{20}{c}} {{C_a} - {C_{as}}} \\ {T - {T_s}} \\ {{T_j} - {T_{js}}} \end{array}} \right]$$, the input (u) $$u=\left[ {\begin{array}{*{20}{c}} {{u_1}} \\ {{u_2}} \end{array}} \right]=\left[ {\begin{array}{*{20}{c}} {{F_{jf}} - Fjfs} \\ {{T_{jf}} - {T_{jfs}}} \end{array}} \right]$$and outputs (y)

$$y=\left[ {\begin{array}{*{20}{c}} {{y_1}} \\ {{y_2}} \end{array}} \right]=\left[ {\begin{array}{*{20}{c}} {T - {T_s}} \\ {{T_j} - {T_{js}}} \end{array}} \right]$$. Here, the values at steady state are specified by the subscript (s) and Eqs. (1,2,5) are linearized around it. The Jacobian matrix formed with these parameters are represented as follows

6 $$\begin{gathered} A=\left[ {\begin{array}{*{20}{c}} {{a_{11}}}&{{a_{12}}}&{{a_{13}}} \\ {{a_{21}}}&{{a_{22}}}&{{a_{23}}} \\ {{a_{31}}}&{{a_{32}}}&{{a_{33}}} \end{array}} \right]= \hfill \\ \left[ {\begin{array}{*{20}{c}} { - \frac{{{F_s}}}{V} - \left( {\exp \left( { - {E_a}/R{T_s}} \right)} \right){k_0}}&{ - \left( {\exp \left( { - {E_a}/R{T_s}} \right)} \right){k_0}{C_{as}}\left( {{E_a}/RT_{s}^{2}} \right)}&0 \\ {\left( {\exp \left( { - {E_a}/R{T_s}} \right)} \right){k_0}\frac{{\left( { - \Delta H} \right)}}{{\rho {C_p}}}}&{ - \frac{F}{V}+\left( {\exp \left( { - {E_a}/R{T_s}} \right)} \right){k_0}\frac{{\left( { - \Delta H} \right)}}{{\rho {C_p}}}{C_{as}}\left( {{E_a}/RT_{s}^{2}} \right) - \frac{{U{A_r}}}{{V\rho {C_p}}}}&{\frac{{U{A_r}}}{{{C_p}V\rho }}} \\ 0&{\frac{{U{A_r}}}{{{C_{pj}}{V_j}{\rho _j}}}}&{ - \left( {\frac{{U{A_r}}}{{{C_{pj}}{V_j}{\rho _j}}}+\frac{{{F_{jfs}}}}{{{V_j}}}} \right)} \end{array}} \right]. \hfill \\ \end{gathered}$$

The input matrix B elements are

7 $$B=\left[ {\begin{array}{*{20}{c}} {{B_{11}}} \\ {{B_{21}}} \\ {{B_{31}}} \end{array}\begin{array}{*{20}{c}} {{B_{12}}} \\ {{B_{22}}} \\ {{B_{32}}} \end{array}} \right]=\left[ {\begin{array}{*{20}{c}} 0 \\ 0 \\ {\frac{{\left( { - {T_{js}}+{T_{jfs}}} \right)}}{{{V_j}}}} \end{array}\begin{array}{*{20}{c}} 0 \\ 0 \\ {\frac{{{F_{jfs}}}}{{{V_j}}}} \end{array}} \right].$$

The elements of output matrix (C) and feedforward matrix (D) are represented as

8 $$\begin{gathered} C=\left[ \begin{gathered} \begin{array}{*{20}{c}} 0&1&0 \end{array} \hfill \\ \begin{array}{*{20}{c}} 0&0&1 \end{array} \hfill \\ \end{gathered} \right] \hfill \\ D=\left[ {\begin{array}{*{20}{c}} 0 \\ 0 \end{array}} \right]. \hfill \\ \end{gathered}$$

By using system matrix (A), input matrix (B), output matrix (C) and feedforward matrix (D), the transfer function of NCSTR is obtained as

9 $${G_p}(s)=C{(sI - A)^{ - 1}}B+D.$$

Furthermore, the steady-state solution is achieved when the derivative of Eq. (1) is set to zero as

10 $$\frac{{d{C_a}}}{{dt}}=\frac{F}{V}\left( {{C_{af}} - {C_a}} \right) - {k_0}\exp \left( {\frac{{ - {E_a}}}{{RT}}} \right){C_a}=0$$

And Eq. (2) is set to 0 as

11 $$\frac{{dT}}{{dt}}=\left( {{T_f} - T} \right)\frac{F}{V}+\left( {\frac{{{k_0} - \Delta H}}{{{C_\rho }}}} \right)\exp \left( {\frac{{ - {E_a}}}{{RT}}} \right)\frac{{{C_a}}}{\rho } - \frac{U}{{{C_p}}}\left( {T - {T_j}} \right)\frac{{{A_r}}}{{V\rho }}=0$$

Solving Eq. (10) for concentration (steady-state) as a function of Temperature (steady-state) will result in

12 $${C_{as}}=\frac{{\frac{{{F_s}}}{V}\left( {{C_{afs}}} \right)}}{{({{{F_s}} \mathord{\left/ {\vphantom {{{F_s}} {V)+{k_0}\exp \left( {\frac{{ - {E_a}}}{{R{T_s}}}} \right)}}} \right. \kern-0pt} {V)+{k_0}\exp \left( {\frac{{ - {E_a}}}{{R{T_s}}}} \right)}}}}$$

The subscript s specifies the steady-state value. Solving Eq. (11) for jacket temperature (steady-state) as a function of reactor Temperature and concentration (steady-state) will result in

13 $${T_{js}}={T_s}+\left[ {{F_s}\rho {C_p}\left( { - {T_{fs}}+{T_s}} \right) - ( - \Delta H)V{k_0}\exp \left( {\frac{{ - {E_a}}}{{R{T_s}}}} \right){C_{as}}} \right].\frac{1}{{U{A_r}}}$$

For a nominal jacket make-up flowrate of 800 ft^3^/h, the steady state operating condition are considered as ${\operatorname{T} _s}=101.{1^o}F$and ${\operatorname{C} _{as}}=0.066lbmol/f{t^3}$^27,28^ and using the operating parameters and constants of NCSTR as described in Table 1, the linearized state space model is obtained as

14 $$A=\left[ {\begin{array}{*{20}{c}} { - 7.9909}&{ - 0.013674}&0 \\ {2922.9}&{4.5564}&{1.4582} \\ 0&{4.7482}&{ - 5.8977} \end{array}} \right],B=\left[ {\begin{array}{*{20}{c}} 0 \\ 0 \\ { - 3.2558} \end{array}} \right],C=\left[ \begin{gathered} \begin{array}{*{20}{c}} 0&1&0 \end{array} \hfill \\ \begin{array}{*{20}{c}} 0&0&1 \end{array} \hfill \\ \end{gathered} \right],D=\left[ {\begin{array}{*{20}{c}} 0 \\ 0 \end{array}} \right].$$

**Table 1:** NCSTR operating parameters and constants.

$\text{E} _{a} = 32,400 Btu/lbmol$	${\operatorname{rC} _r}=53.25Btu/f{t^2}{}^{o}F$
$\text{V} = 85ft^{3}$	${\operatorname{C} _{af}}=0.132lbmol/f{t^3}$
$- DH = 39000Btu/lbmol$	$\operatorname{F} =340f{t^3}/hr$
$\text{k} _{0} = 16.96 \times 10^{{12}} hr^{{ - 1}}$	${r_j}{C_{rj}}=55.6Btu/f{t^3}{}^{o}F$
$\text{V} /F = 0.25hr$	${{T} _f}=6{0^\circ }F$
$\text{T} _{{jf}} = 0^{o} F$	$\operatorname{R} =1.987Btu/lbmol{}^{o}F$
$\text{UA} = 6600Btu/hr{}^{o}F$	${\operatorname{V} _j}/V=0.25$

Placing the matrix A, B, C and D in Eq. (9), the transfer function of the NCSTR is obtained as:

15 $${G_p}(s)=\frac{T}{{{F_{jf}}}}=\frac{{ - 4.747s - 37.94}}{{{s^3}+9.332{s^2}+16.89s - 34.35}},$$

When the jacket makeup flowrate is changed to 270.105 *ft*^*3*^*/hr*, the NCSTR model is obtained as

16 $${G_p}(s)=\frac{T}{{{T_{jf}}}}=\frac{{15.75s+125.9}}{{{s^3}+18.99{s^2}+50.05s+0}}.$$

The poles of this model indicate integrating dynamics located at 0, − 3.16 and − 15.82. When jacket inlet temperature is considered as manipulating variable, the transfer function of the NCSTR is obtained as

17 $${G_p}(s)=\frac{T}{{{T_{jf}}}}=\frac{{46.66s+372.9}}{{{s^3}+40.18{s^2}+122.8s+75.4}}.$$

The poles of the model in Eq. (17) have three stable poles at -0.84, -2.43 and − 36.9.

Equations (15), (16) and (17), indicates that the dynamic behaviour of NCSTR varies under various operating conditions. And literature shows, most of the researchers have controlled NCSTR by using stable third order model with or without integrating dynamics^29–32^. However, considering the unstable NCSTR model for controller synthesis is quite cumbersome and as per author’s best knowledge, no work exists that handles the unstable NCSTR through parallel control structure (as in Eq. (15)) in PCCS. Hence, in this work, unstable third order NCSTR model is used to design the controller of PCCS as it captures better dynamics of NCSTR, provides better control on the concentration of the reactants in the reactor, maintains constant flow output even in the presence of disturbances.

## Controller design of PCCS

This section presents the controller design approach for secondary loop and primary loop of the parallel cascade control structure.

**Fig. 2:**
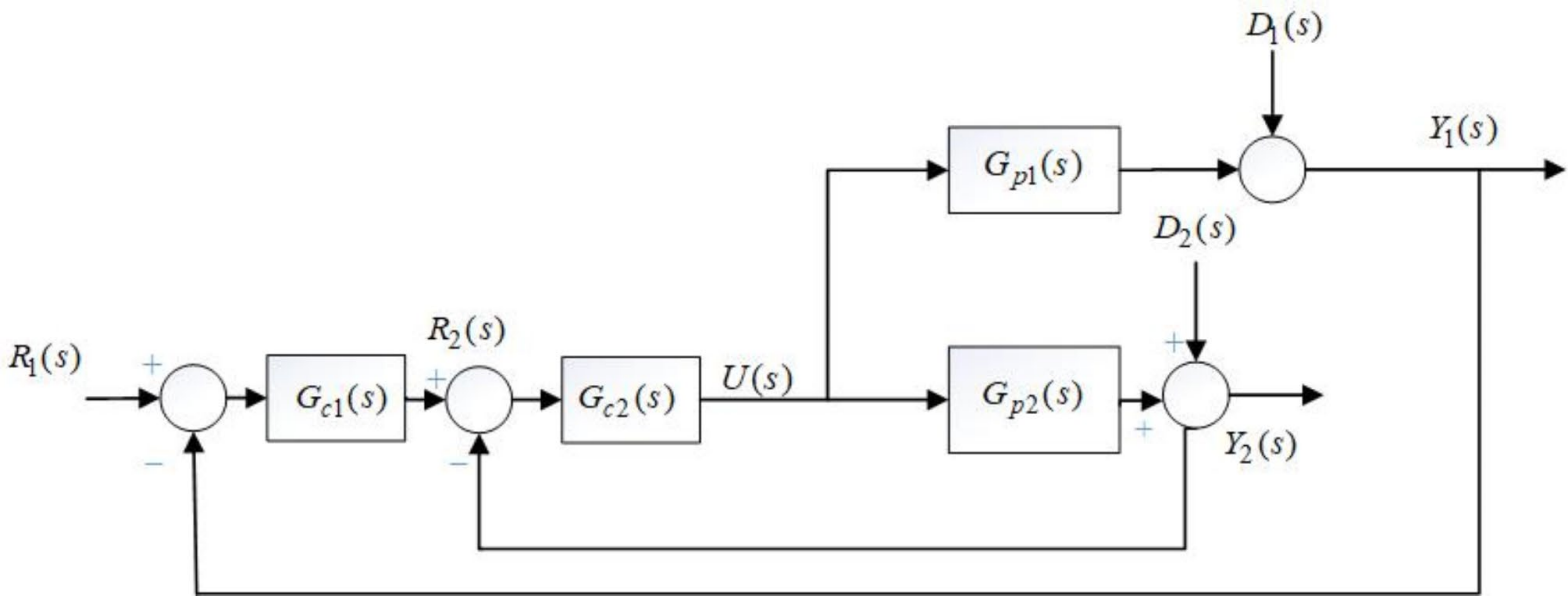
Block diagram of PCCS.

**Fig. 3:**
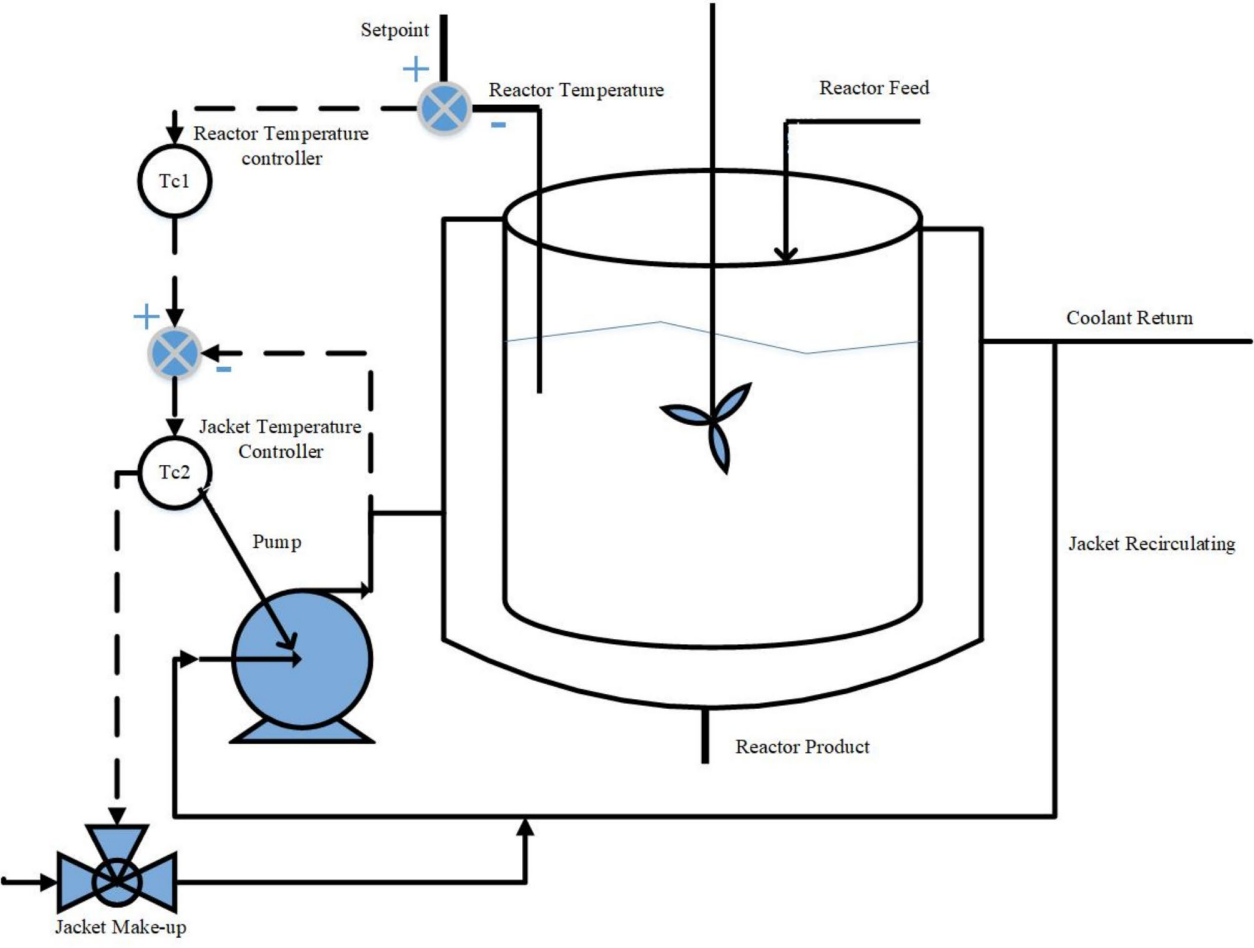
Process flow diagram for NCSTR in a PCCS.

The PCCS is presented in Fig. 2, where *R*_*1*_*(s)* and *R*_*2*_*(s)* are the inputs to the primary loop and secondary loop, respectively. *Y*_*1*_*(s)* is the final output i.e. reactor temperature and *Y*_*2*_*(s)* is the output of secondary loop i.e. jacket temperature. The load disturbances entering into the system are indicated by *D*_*1*_*(s)* and *D*_*2*_*(s). G*_*c1*_*(s)* and *G*_*c2*_*(s)* are the controllers used to control primary loop plant *G*_*p1*_*(s)* and secondary loop plant *G*_*p2*_*(s).* In this work, the controllers *G*_*c2*_*(s)* and *G*_*c1*_*(s)* are considered as:

16 $${G_{c2}}(s)={K_{pil}}+\frac{{{K_{iil}}}}{s},$$

17 $${G_{c1}}(s)={K_{pout}}+\frac{{{K_{iout}}}}{s}+{K_{dout}},$$

here, the proportional gains are *K*_*pil*_ and *K*_*pout*_, integral and derivative gains are represented by *K*_*iil*_, *K*_*iout*_ and *K*_*dout*_, respectively. The process flow diagram of NCSTR in a PCCS is shown in Fig. 3.

To design the controller for PCCS, the model of the NCSTR mentioned in Eq. (15) is considered ${G_p}(s)=\frac{T}{{{F_{jf}}}}=\frac{{ - 4.747s - 37.94}}{{{s^3}+9.332{s^2}+16.89s - 34.35}}$.

In Eq. (15), the make-up flowrate in jacket act as manipulating variable and has two stable poles at$-5.25 \pm 1.27i$ and one unstable pole at + 1.175.

Equation (15) can be further realized as

18 $${G_p}(s)=\frac{T}{{{F_{jf}}}}=\frac{{ - 4.747s - 37.94}}{{(s+(5.25+1.27i))*(s+(5.25 - 1.27i))*(s - 1.175)}}$$

Equation (18) is decomposed into two parts as

19 $${G_p}(s)=\frac{{ - 4.747s - 34.94}}{{(s - 1.175)}}*\frac{1}{{({s^2}+10.51s+25.99)}}$$

In this work, the inner loop dynamics are considered as first order unstable with inverse response whereas the primary loop dynamics are considered as stable second order system and is represented as.

20 $${G_{p2}}(s)=\frac{{ - 4.747s - 34.94}}{{s - 1.175}} and {G_{p1}}=\frac{1}{{{s^2}+10.51s+25.99}}.$$

The load disturbances are mainly associated with the inner loop, so this loop is designed with an objective to eliminate disturbance and to achieve good regulatory performance. The DCLM_FLD is selected that comprises of predicted performance requirements for inner loop. Based on DCLM_FLD, the inner loop controller is synthesized with a purpose to force the inner loop trajectory in such a manner that it tracks the defined disturbance rejection trajectory. To attain the said target, the perception of matching the DCLM_FLD with designed transfer function for load disturbance (DTF_FLD) has been utilized in frequency domain. Subsequently, the controller in the outer loop is synthesized by taking into account the inner loop controller and plant as a component of the outer loop process. A DCLM_FST is considered and the controller is synthesized to follow the desired setpoint tracking performance prerequisites by model matching between DCLM_FST and designed transfer function for setpoint tracking (DTF_FST). The comprehensive design details for the controller of inner loop *G*_*c2*_*(s)* and outer loop *G*_*c1*_*(s)* are summarized as follows:

### Design of inner/secondary loop controller

The controller of inner loop *G*_*c2*_*(s)* is synthesized to reduce the consequence of the disturbances entering into *T*_*jf*_. In this vein, the first step is to consider the DCLM_FLD as

21 $$DCLM\_FLD=\frac{{{K_0}.s}}{{{{(1+{\lambda _{il}}s)}^{^{{_{{{n_{il}}}}}}}}}}$$

Here, the DCLM_FLD comprises of one zero at origin so that load disturbance signal can be minimized. Furthermore, the DCLM_FLD have tuning parameter${\lambda _{il}}$that decide the speed of the closed loop system and helps in providing the trade-off between performance and robustness. ${n_{il}}$is the power of the DCLM_FLD and ${K_0}$is equal to *1/K*_*iil*_^2,33^.

After choosing the DCLM_FLD, the controller of the inner loop is synthesized to match the performance of the closed-loop model with DCLM_FLD. To accomplish this, closed-loop model DTF_FLD and DCLM_FLD is matched which is mathematically represented as

22 $${\left. {DTF\_FLD\left( {\frac{{{\text{ }}{Y_2}(s)}}{{{\text{ }}{D_2}(s)}}} \right)} \right|_{_{{s=j{\omega _n}}}}}={\left. {\frac{{{G_{p2}}(s)}}{{1+{G_{p2}}(s){G_{c2}}(s)}}} \right|_{_{{s=j{\omega _n}}}}} \cong {\left. {DCLM\_FLD(s)} \right|_{_{{s=j{\omega _n}}}}}$$

Equation (22) is re-arranged as

23 $${\left. {{G_{c2}}(s)} \right|_{s=j{\omega _n}}}={\left. {\frac{1}{{DCLM\_FLD(s)}} - \frac{1}{{{G_{p2}}(s)}}} \right|_{s=j{\omega _{_{n}}}}}={\left. {{C_{ont}}~(s)} \right|_{s=j{\omega _{_{n}}}}}~~~~~~~~n={\text{ }}1,{\text{ }}2,{\text{ }}3 \ldots .$$

A perfect execution of the inner loop controller *G*_*c2*_*(s)* as in Eq. (23) would yield response of *D* similar to that of DCLM_FLD, but the corresponding *G*_*c2*_*(s*) controller might be inappropriate for practical or industrial implementation. The reason is, complicated orientation of the controller structure may not practically attainable. Usually in literature, researchers use model having low order and several approximation approach such as pade series, Taylor series, Maclaurin series approximation, etc. to approximate the controller into PI or PID structure. For model having higher order dynamics, researchers generally approx. them into low order model to apply their design method. Although, in the approach suggested hereunder, the controller is obtained in PI or PID form by using the concept of model matching in low frequency region. Matching in lower frequency domain helps us to apply the proposed method on any order system with approximating them into lower order and also devoid us from time delay approximation^2,20,21,34^.

Substituting *G*_*c2*_ (Eq. 16) in Eq. (23), the resulting expression is achieved

24 $${K_{pil}}+\frac{{{K_{iil}}}}{{j{\omega _n}}}=\frac{{{K_{iil}}{{({\lambda _{il}}(j{\omega _n})+1)}^{^{{_{{{n_{il}}}}}}}}}}{{(j{\omega _n})}} - \frac{1}{{{G_{p2}}(j{\omega _n})}}.$$

Assuming $\frac{{{K_{iil}}{{({\lambda _{il}}(j{\omega _n})+1)}^{^{{_{{{n_{il}}}}}}}}}}{{(j{\omega _n})}}={a_{il}}$and$\frac{1}{{{G_{p2}}(j{\omega _n})}}={b_{il}}$, Eq. (24) is re-written as

25 $${K_{pil}}+\frac{{{K_{iil}}}}{{j{\omega _n}}}={K_{iil}}\left( {R\left[ {{a_{il}}} \right]+jI[{a_{il}}]} \right) - \left( {R\left[ {{b_{il}}} \right]+jI[{b_{il}}]} \right),$$

here, *I* and *R* represents the imaginary and real parts of Eq. (25), respectively.

Dissociating the *R* and the *I* components from Eq. (25) results in

26 $$\begin{gathered} {K_{pil}} - {K_{iil}}\operatorname{R} [{a_{il}}]= - \operatorname{R} [{b_{il}}] \\ \left( {\frac{{ - 1}}{{{\omega _n}}} - \operatorname{I} [{a_{il}}]} \right){K_{iil}}= - \operatorname{I} [{b_{il}}]. \\ \end{gathered}$$

As the *G*_*c2*_ is selected as PI controller, the frequency point (${\omega _n}$) is selected as ${\omega _1}$whose value is in close proximity to 0.1% of the total bandwidth of DCLM_FLD. The complete explanation concerning the selection of ${\omega _n}$is clarified in^2,20,21,34^. For lucidity, Eq. (26) is arranged in matrix form as

27 $$\left[ {\begin{array}{*{20}{c}} 1&{ - \operatorname{R} [{a_{il}}({\omega _1})]} \\ 0&{\left( {\frac{{ - 1}}{{{\omega _1}}} - \operatorname{I} [{a_{il}}({\omega _1})]} \right)} \end{array}} \right]\left[ {\begin{array}{*{20}{c}} {{K_{pil}}} \\ {{K_{iil}}} \end{array}} \right]=\left[ {\begin{array}{*{20}{c}} { - \operatorname{R} [{b_{il}}({\omega _1})]} \\ { - \operatorname{I} [{b_{il}}({\omega _1})]} \end{array}} \right]$$.

By computing Eq. (27), the inner loop controller G_c2_ parameters are determined.

### Design of outer/primary loop controller

The outer loop controller is designed with an objective to achieve and track desired reactor temperature. While synthesizing the controller of the outer loop, the inner/secondary loop is deemed as a portion of the outer loop plant. The DTF_SFT obtained from Fig. 2 is expressed as

28 $$DTF\_SFT(s)=\frac{{{G_{c2}}(s){G_{c1}}(s){G_{p1}}(s)}}{{1+{G_{c2}}(s)\left( {{G_{p2}}(s)+{G_{p1}}(s){G_{c1}}(s)} \right)}}$$

The DCLM_FST is selected as

29 $$DCLM\_FST(s)=\frac{{( - 4.74s - 37.94)\left( {\gamma s+1} \right)}}{{{{({\lambda _{ol}}s+1)}^{{n_{ol}}}}}}.$$

Here, right hand zero is considered in DCLM_FST as the outer loop controller cannot eliminate the effect of it. ${\lambda _{ol}}$and${n_{ol}}$are the outer loop tuning parameter and the order of the DCLM_FST, respectively. $(\gamma s+1)$ is considered in the DCLM_FST to prevent the cancellation between unstable pole of the process and zero of the controller.

The next step involves the selection of the DCLM_FST pole $s= - {1 \mathord{\left/ {\vphantom {1 {{\lambda _{ol}}}}} \right. \kern-0pt} {{\lambda _{ol}}}}$ so as to make it the pole of the entire system in order to achieve the anticipated transient performance. When the DCLM_FST pole is placed in the characteristic equation (Eq. 28), a connection between secondary plant, primary plant, secondary controller and primary controller is established as

30 $${K_{pout}} - {\lambda _{ol}}{K_{iout}} - {K_{dout}}/{\lambda _{ol}}={\left. { - \left( {{{\left[ {1+{G_{c2}}(s){G_{p2}}(s)} \right]} \mathord{\left/ {\vphantom {{\left[ {1+{G_{c2}}(s){G_{p2}}(s)} \right]} {\left. {\left[ {{G_{p1}}(s){G_{c2}}(s)} \right]} \right|}}} \right. \kern-0pt} {\left. {\left[ {{G_{p1}}(s){G_{c2}}(s)} \right]} \right|}}} \right)} \right|_{_{{s= - 1/{\lambda _{ol}}}}}}={X_{ol}}$$

As per Eq. (30), the properly placed pole of DCLM_FST results in the desired transient performance, whereas, the enhanced steady state performance is achieved by frequency response matching between DTF_SFT and DCLM_FST. Mathematically, the matching between DTF_SFT and DCLM_FST is written as

31 $$\begin{gathered} DTF\_SFT~~~~~~~~~~~~~~~~~~~~~~~~~~~~~~~~~~~~~~~~~~~~~~~~ \cong DCLM\_FST \hfill \\ {\left. {\frac{{{G_{c2}}(s){G_{c1}}(s){G_{p1}}(s)}}{{1+{G_{c2}}(s)\left( {{G_{p2}}(s)+{G_{p1}}(s){G_{c1}}(s)} \right)}}} \right|_{s=j{\omega _p}}} \cong {\left. {\frac{{( - 4.74s - 37.94)\left( {\gamma s+1} \right)}}{{{{({\lambda _{ol}}s+1)}^{{n_{ol}}}}}}} \right|_{_{{s=j{\omega _p}}}}} \hfill \\ \end{gathered}$$

Arranging Eq. (31) for *G*_*c1*_*(s)* yields

32 $${\left. {{G_{c1}}(s)} \right|_{s=j{\omega _p}}}={\left. {\frac{{DCLM\_FST\left( {1+{G_{p2}}{G_{c2}}} \right)}}{{{G_{p1}}{G_{c2}}\left( {1 - DCLM\_FST} \right)}}} \right|_{s=j{\omega _p}}}$$

Substituting DCLM_FST in Eq. (32) yields

33 $${\left. {{G_{c1}}(s)} \right|_{s=j{\omega _p}}}={\left. {\frac{{( - 4.74s - 37.94)\left( {\gamma s+1} \right)\left( {1+{G_{p2}}{G_{c2}}} \right)}}{{{G_{p1}}{G_{c2}}\left( {{{({\lambda _{ol}}s+1)}^{{n_{ol}}}} - ( - 4.74s - 37.94)\left( {\gamma s+1} \right)} \right)}}} \right|_{s=j{\omega _p}}}$$

To provide internal stability, the term ${\lambda _{ol}}$ and $\gamma$ in the denominator of Eq. (33) are chosen as follows

34 $${\left. {\left( {{{({\lambda _{ol}}s+1)}^{{n_{ol}}}} - ( - 4.74s - 37.94)\left( {\gamma s+1} \right)} \right)} \right|_{s=1/{\tau _u}}}=0$$

Here, $s=1/{\tau _u}$is the unstable pole of the NCSTR. So, the pole is placed at *s = 1/1.175* and after some manipulation, $\gamma$is calculated as

35 $$\gamma ={\left. {\frac{{{{\left( {{\lambda _{ol}}s+1} \right)}^{{n_{ol}}}}}}{{\left( { - 4.74s - 37.94} \right)s}} - \frac{1}{s}} \right|_{s=1/1.175}}$$

Assuming$\frac{{DCLM\_FST\left( {1+{G_{p2}}{G_{c2}}} \right)}}{{{G_{p1}}{G_{c2}}\left( {1 - DCLM\_FST} \right)}}=Z$, the subsequent equation is achieved as

36 $${K_{pout}}+j\left( {\frac{{{K_{iout}}}}{{{\omega _p}}}+{K_{dout}}{\omega _p}} \right)=real\left( Z \right)+j\left( {imag\left( Z \right)} \right)$$

Equation (36) is decomposed into two sub-equations as

37 $$\begin{gathered} {K_{pout}}=R\left[ Z \right] \\ \left( {\frac{{ - 1}}{{{\omega _p}}}} \right){K_{iout}}+{\omega _p}{K_{dout}}=I\left[ Z \right]. \\ \end{gathered}$$

For simplicity, Eqs. (37) and (30) are categorized in matrix form as

38 $$\left[ {\begin{array}{*{20}{c}} 1&{ - {\lambda _{ol}}}&{ - 1/{\lambda _{ol}}} \\ 1&0&0 \\ 0&{\left( {\frac{{ - 1}}{{{\omega _p}}}} \right)}&{{\omega _p}} \end{array}} \right]\left[ {\begin{array}{*{20}{c}} {{K_{pout}}} \\ {{K_{iout}}} \\ {{K_{dout}}} \end{array}} \right]=\left[ {\begin{array}{*{20}{c}} {{X_{ol}}} \\ {R\left[ Z \right]} \\ {I\left[ Z \right]} \end{array}} \right].$$

**Fig. 4:**
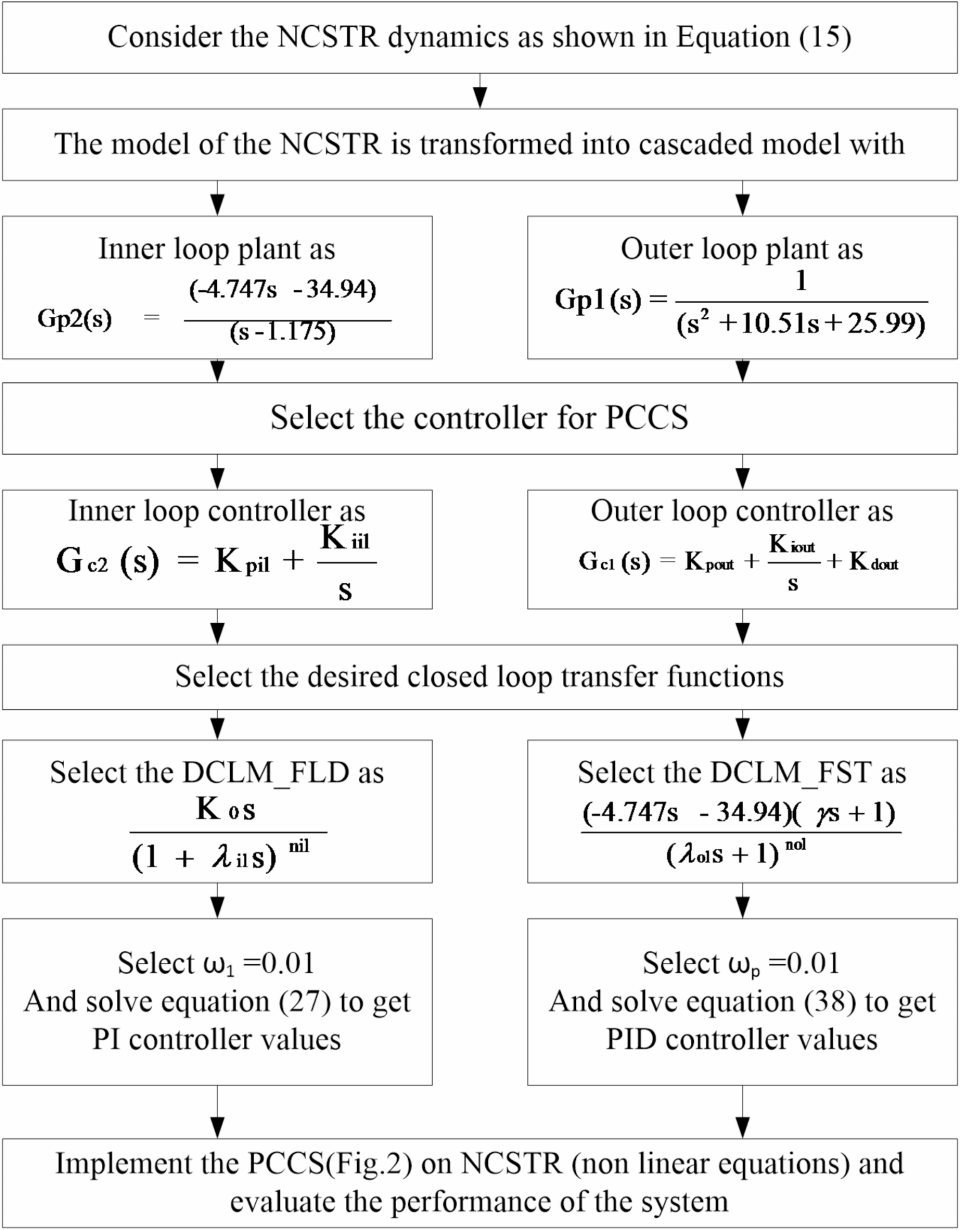
Steps involved in the proposed PCCS to control NCSTR.

The solution of Eq. (38) will yield the primary controller parameters value, thereby completing the design of PCCS controllers. For summary and clear demonstration, the steps required to design the PCCS controllers are presented in Fig. 4.

**Fig. 5:**
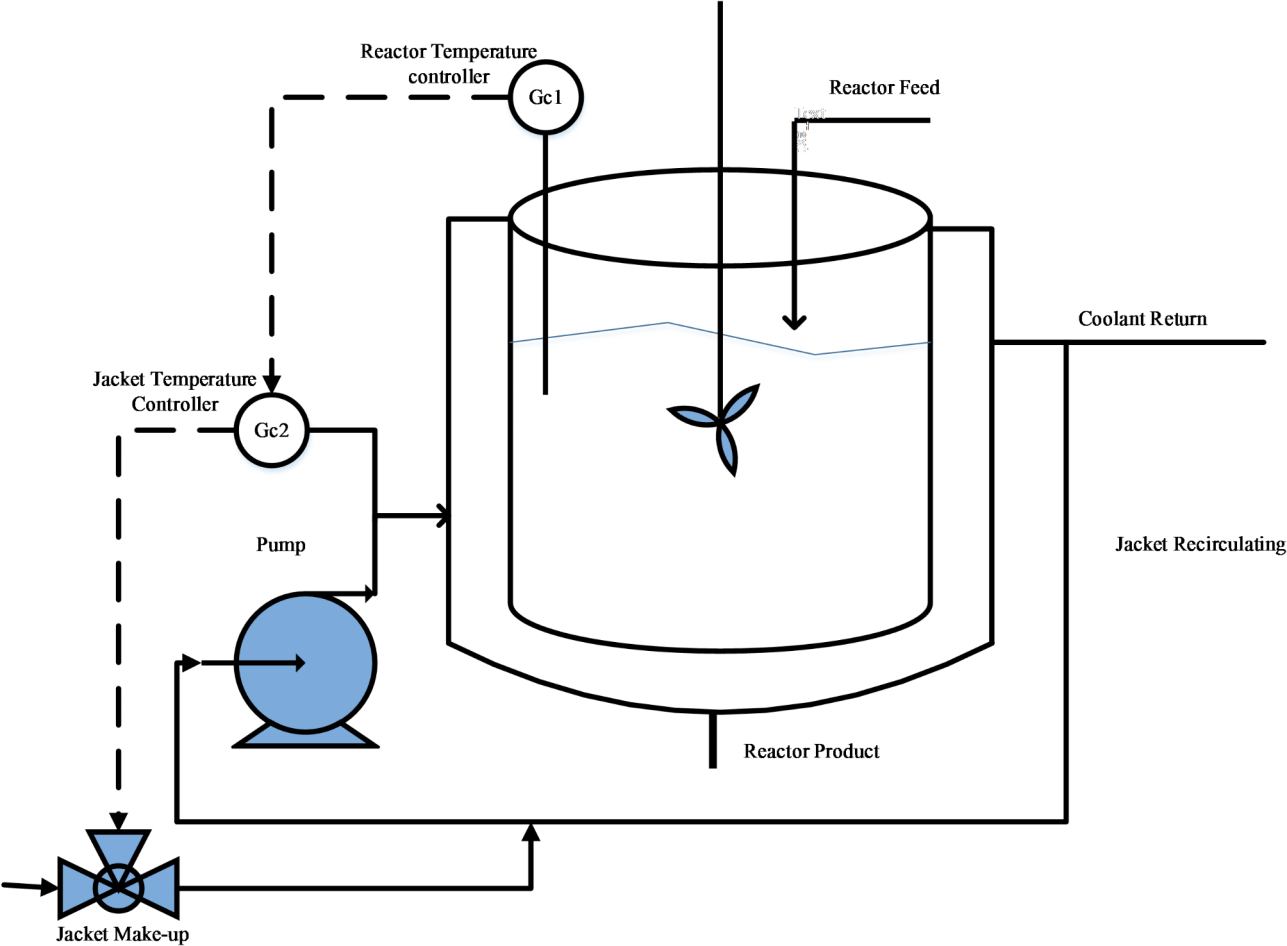
Process flow diagram for CSTR in a CCS.

The proposed system is compared with the cascade control structure and parallel control structure as mentioned in the literature^20,21,35^. The excerpts of the methods proposed in^20,21,35^ are as follows:

For cascade control structure (CCS), a direct synthesis (DS) method in the frequency domain for load disturbance rejection and set-point tracking has been used. This method is able to controls the open-loop unstable process in the primary loop and the open-loop unstable, integrating and stable processes in the secondary loop of the CCS. This method has been satisfactorily applied on the unstable third order CSTR model. For more insight about the method, please refer to^20,21^. The NCSTR is controlled in CCS as shown in the Fig. 5.

**Fig. 6:**
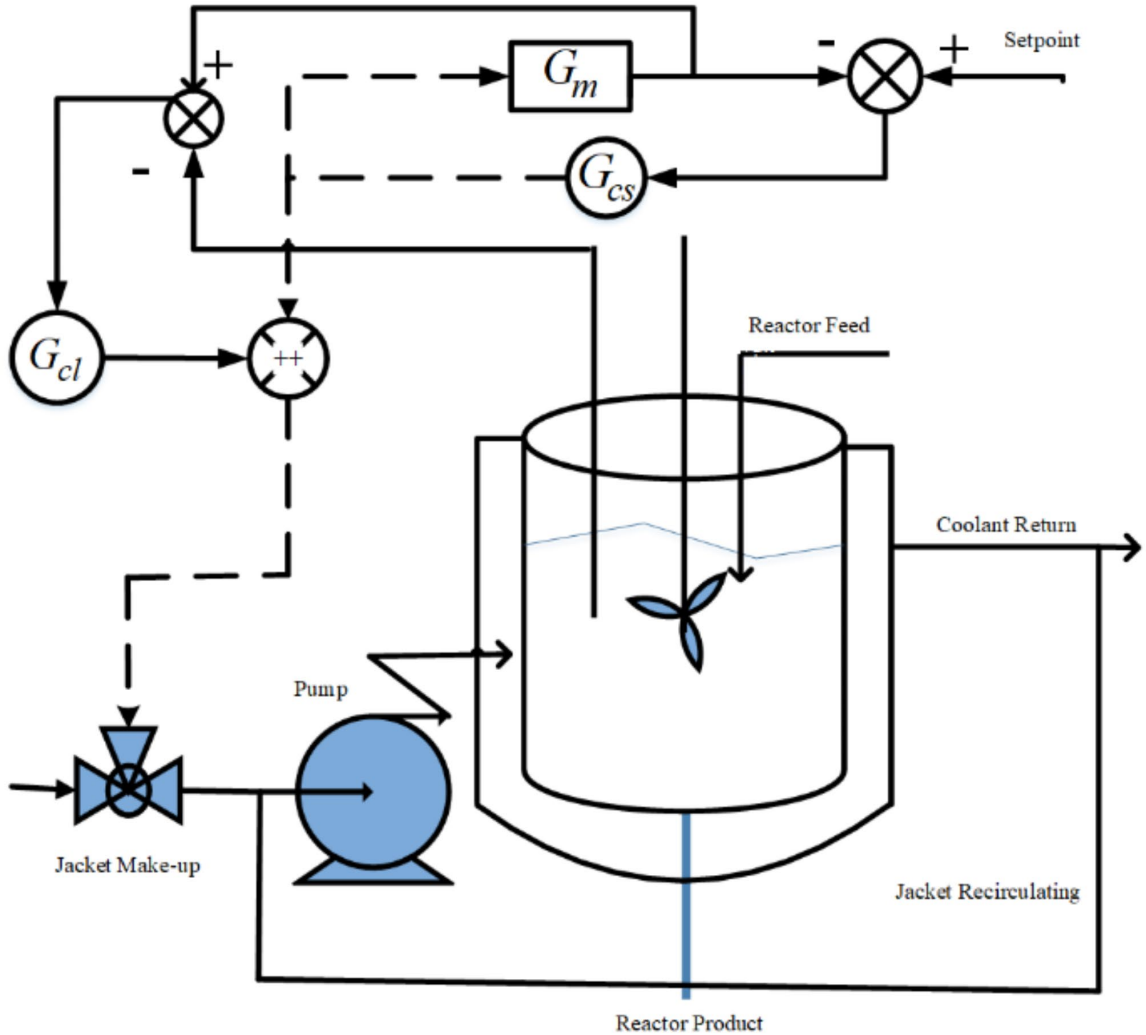
Schematic diagram of CSTR controlled through PCS.

For parallel control configuration, a direct synthesis approach and frequency response matching for better setpoint tracking and enhanced load disturbance rejection. Detail about the method is mentioned in^20,35^, where the method is applied on stable and integrating process of any order having diverse dynamics. In this work, to apply this method on the unstable third order CSTR model, the authors have used the desired model for the setpoint in the form of${M_{r,y}}(s)=\frac{{(\beta s+1)}}{{{{({\lambda _s}s+1)}^{{n_1}}}}}$ and for load disturbance the desired model selected is having the form of ${M_{d,y}}=\frac{{sK}}{{{{(\lambda s+1)}^{^{{_{n}}}}}}}$. Now, model matching is performed between the desired model and designed model at a low frequency region to synthesize the PIDF controller. The NCSTR is controlled in PCS as shown in the Fig. 6.

From the aforementioned methods, the controller setting obtained is tabulated in the Table 3.

## Robustness analysis

To check robustness of the control system, a simple, efficient and commonly applied method is Kharitonov’s theorem^36^. In this theorem, the complete family of the polynomial represented by the characteristic equation is checked for robustness by analyzing the movement of the polygon through n quadrant in the complex plane along with Hurwitz stability analysis of the four Kharitonov polynomials.

The entire family of the polynomial$\:\:K(s,l)$ is expressed as

39 $$P=\left\{ {\left. {K\left( {s,l} \right)=\sum\limits_{{j=0}}^{m} {{l_j}{s^j},l \in \left[ {l_{j}^{{\hbox{min} }},l_{j}^{{\hbox{max} }}} \right]} ,j=0,1,\ldots.,m} \right\}} \right..$$

Here, the parameter *l* defines uncertainty with upper and lower uncertainty bound of the plant as $l_{j}^{{\hbox{min} }}$and $l_{j}^{{\hbox{max} }}$, respectively.

As per Kharitonov’s theorem, the complete family of polynomial is said robustly stable if the subsequent four Kharitonov’s polynomials mentioned hereunder are stable

40 $${P_1}(s)=l_{0}^{{\hbox{min} }}+l_{1}^{{\hbox{min} }}s+l_{2}^{{\hbox{max} }}{s^2}+l_{3}^{{\hbox{max} }}{s^3}+l_{4}^{{\hbox{min} }}{s^4}+\ldots.$$

41 $${P_2}(s)=l_{0}^{{\hbox{max} }}+l_{1}^{{\hbox{max} }}s+l_{2}^{{\hbox{min} }}{s^2}+l_{3}^{{\hbox{min} }}{s^3}+l_{4}^{{\hbox{max} }}{s^4}+\ldots.$$

42 $${P_3}(s)=l_{0}^{{\hbox{max} }}+l_{1}^{{\hbox{min} }}s+l_{2}^{{\hbox{min} }}{s^2}+l_{3}^{{\hbox{max} }}{s^3}+l_{4}^{{\hbox{max} }}{s^4}+\ldots.$$

43 $${P_4}(s)=l_{0}^{{\hbox{min} }}+l_{1}^{{\hbox{max} }}s+l_{2}^{{\hbox{max} }}{s^2}+l_{3}^{{\hbox{min} }}{s^3}+l_{4}^{{\hbox{min} }}{s^4}+\ldots.$$

The Kharitonov’s theorem is decrypted in both the forms geometrically as well as analytically. In geometrical decryption, the boundaries of the rectangle created by the Eqs. (40)–(43) are analyzed. If the boundaries lie away from the origin of the complex plane and the rectangles (of nth-degree interval) moves in a counter-clockwise direction without bisecting or touching the origin through n quadrants, then the considered system is said to be robustly stable. For analytical decryption, the roots of the Kharitonov polynomial mentioned in Eqs. (40)–(43) are to be investigated. If the entire roots lie on the left half of the complex plane, then each and every interval polynomial inside the uncertainty bound $\left[ {l_{j}^{{\hbox{min} }},l_{j}^{{\hbox{max} }}} \right]$is considered to be robustly stable.

The characteristic equation of the system as mentioned in Eq. (28) is given as

44 $$1+{G_{c2}}(s)\left( {{G_{p2}}(s)+{G_{p1}}(s){G_{c1}}(s)} \right)$$

When perturbation of $\pm$90% and $\pm$50% perturbation is introduced in the time constant and gain, respectively, of the inner and outer loop of the PCCS, the following perturbed models are obtained

45 $$\begin{gathered} {G_{p2}}(s)=\frac{{1.5\left( { - 4.747s - 34.94} \right)}}{{1.9\left( {s - 1.175} \right)}},\frac{{0.5\left( { - 4.747s - 34.94} \right)}}{{0.1\left( {s - 1.175} \right)}} \hfill \\ {G_{p1}}=\frac{{1.5}}{{1.9\left( {{s^2}+10.51s+25.99} \right)}},\frac{{0.5}}{{0.1\left( {{s^2}+10.51s+25.99} \right)}} \hfill \\ \end{gathered}$$

By using Eq. (45), the four Kharitonov polynomials are obtained as

46 $${P_1}\left( s \right)=0+0s+{\text{1330800}}{{\text{s}}^2}+{\text{783400}}{{\text{s}}^3}+{\text{51300}}{{\text{s}}^4}+4000{s^5},$$

47 $${P_2}\left( s \right)=100+{\text{ 13920}}s+{\text{164260}}{s^2}+{\text{261150}}{s^3}+{\text{ 443590}}{s^4}+120{s^5},$$

48 $${P_3}\left( s \right)={\text{100}}+{\text{ 4010}}s+{\text{164260}}{s^2}+{\text{783440}}{s^3}+{\text{ 443590}}{s^4}+40{s^5},$$

49 $${P_4}\left( s \right)={\text{400}}+{\text{13900}}s+{\text{51300}}{s^2}+{\text{261200}}{s^3}+{\text{1330800}}{s^4}+{\text{ 100}}{s^5}.$$

**Fig. 7:**
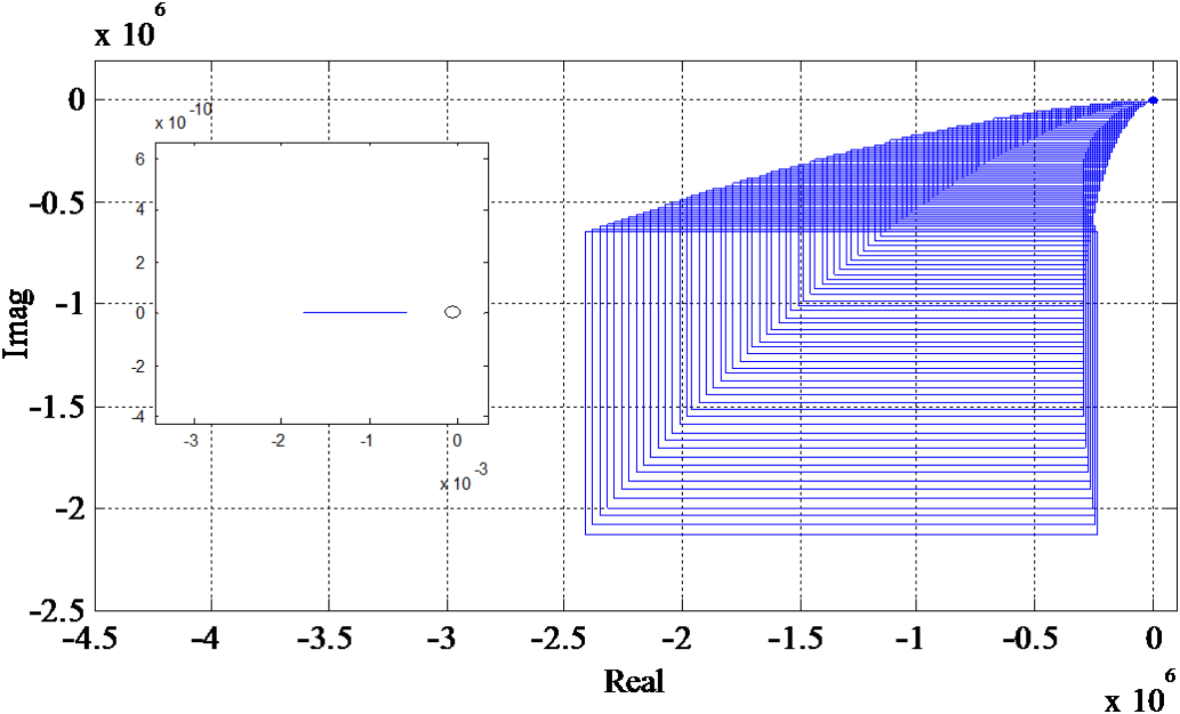
Kharitonov rectangles for Eqs. (46)–(49).

The roots of Eqs. (46)–(49) are tabulated in Table 2. As per analytical interpretation of the theorem it is concluded that the PCCS is robustly stable since the roots lie on the negative half of the complex plane. For stability in geometrical sense, the Kharitonov rectangles are produced by considering Eqs. (46)–(49). The produced Kharitonov rectangles are illustrated in Fig. 7 that clearly shows the counter-clockwise movement of the Kharitonov rectangles. The graph is zoomed to display what is occurring in the vicinity of the origin (0, 0). Since origin is excluded from the rectangles form by the closed-loop characteristic equation of the system, the system is said to be robustly stable. Hence, in both the sense (analytical and geometrical), the PCCS is found to be robustly stable.

**Table 2:** The roots of eqs. (46)–(49) for PCCS.

$P_{1} \left( s \right)$	${P_2}\left( s \right)$	${P_3}\left( s \right)$	${P_4}\left( s \right)$
$$\begin{array}{*{20}l} {~ - 5.4496 + 12.0371i} \hfill \\ {~ - 5.4496 - 12.0371i} \hfill \\ {~ - 1.9002 + 0.0000i} \hfill \\ {~ - 0.0000 + 0.0000i} \hfill \\ {~~0.0000 + 0.0000i} \hfill \\ \end{array}$$	$$\begin{gathered} 1.0e+03 * \hfill \\ \left[ {\begin{array}{*{20}{l}} {-3.7273 + 0.0000i} \\ {~-0.0002 + 0.0005i} \\ {~-0.0002 - 0.0005i} \\ {~-0.0001 + 0.0000i} \\ {~-0.0000 + 0.0000i} \end{array}} \right] \hfill \\ \end{gathered}$$	$$\begin{gathered} 1.0e+04 * \hfill \\ \left[ {\begin{array}{*{20}{l}} {-1.1182 + 0.0000i} \\ {~-0.0002 + 0.0000i} \\ {~-0.0000 + 0.0000i} \\ {~-0.0000 + 0.0000i} \\ {~-0.0000 - 0.0000i} \end{array}} \right] \hfill \\ \end{gathered}$$	$$\begin{gathered} 1.0e+04 * \hfill \\ \left[ {\begin{array}{*{20}{l}} {~-1.1184 + 0.0000i} \\ {~~0.0000 + 0.0000i} \\ {~~0.0000 - 0.0000i} \\ {~-0.0000 + 0.0000i} \\ {~-0.0000 + 0.0000i} \end{array}} \right] \hfill \\ \end{gathered}$$

## Simulation study

In this section, the efficacy of the aforementioned controller design approach for PCCS has been verified through simulation on CSTR differential equations (Eqs. (1,2,5)). The proposed method is compared with different structures like series cascade control structure and parallel control structure (PCS). The controller parameter for these structures and the suggested method are tabulated in Table 3. The performance of PCCS, CCS and PCS have been evaluated on the basis of (i) *IAE*, (ii) *ISE*, (iii) *ITAE*, (iv) settling time for setpoint (*Y*_*1*_), (v) settling time for load disturbance (*D*_*1*_), (vi) settling time for load disturbance (*D*_*2*_) (vii) peak overshoot (*Y*_*max*_) for *Y*_*1*,_*D*_*1*_ and *D*_*2*_.

**Fig. 8:**
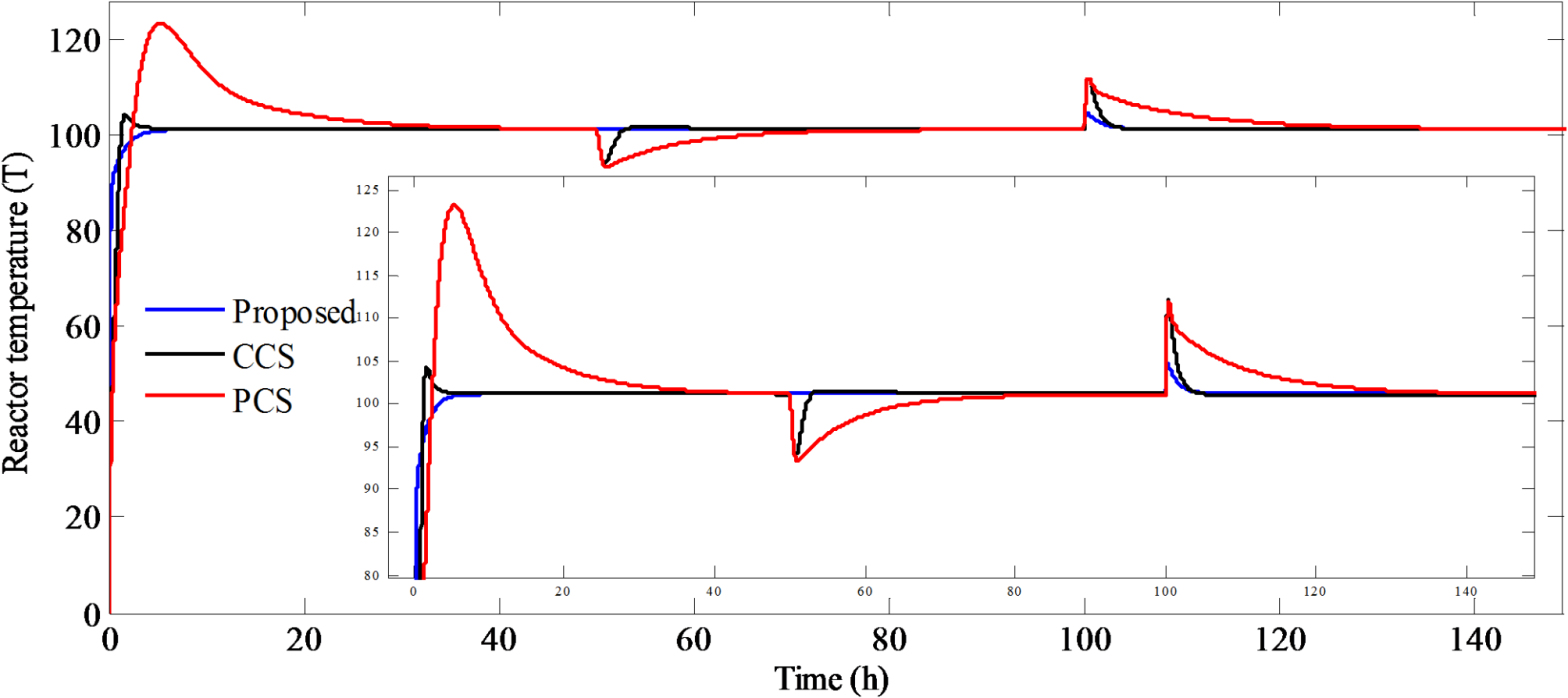
Temperature of the reactor for PCCS, CCS, and PCS.

**Fig. 9:**
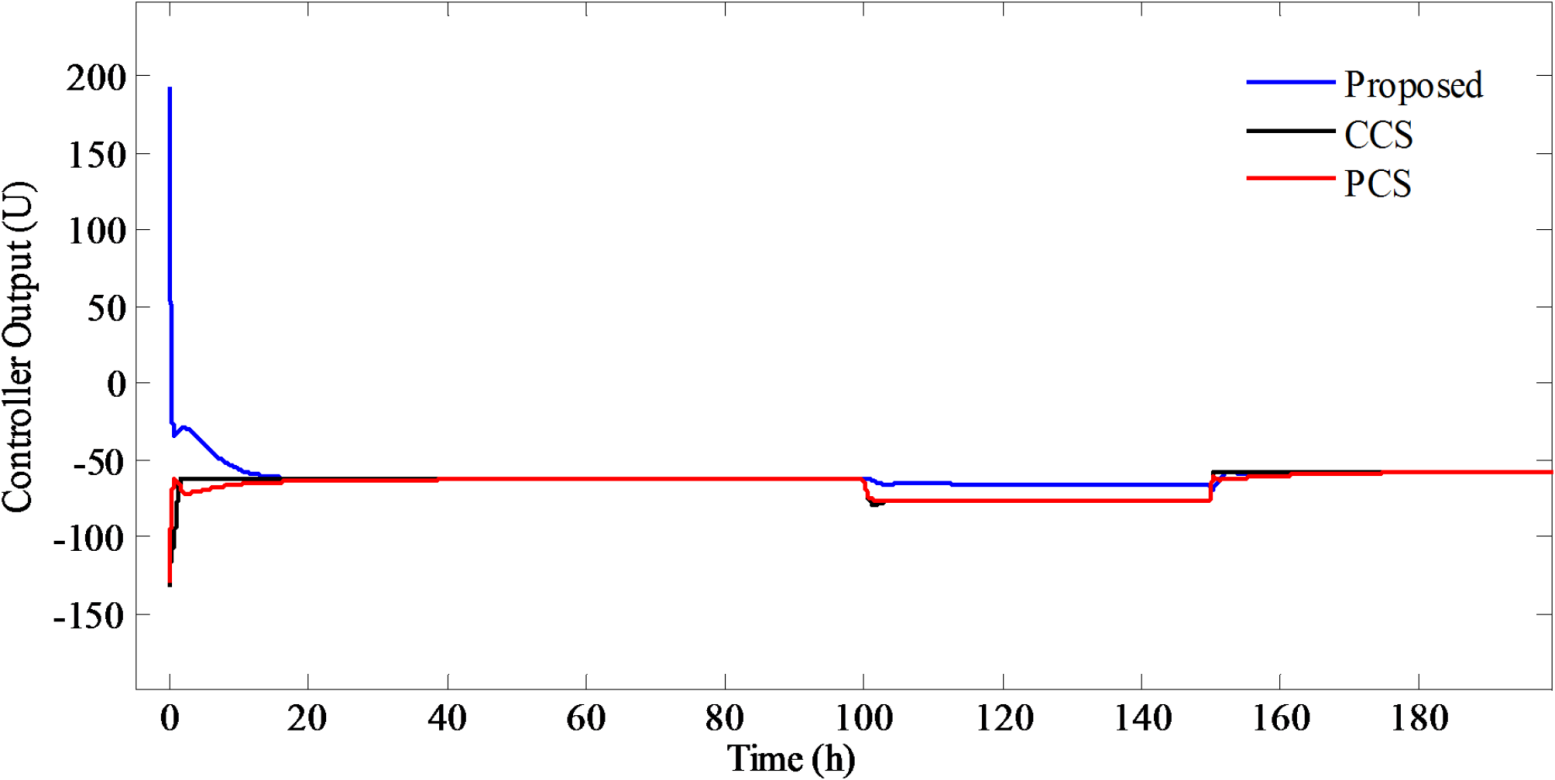
Jacket make-up flowrate for PCCS, CCS, and PCS.

Simulations are performed on the NCSTR (Eqs. (1,2,5) by giving a signal of step input having magnitude of $101.{1^o}F$at time equal to zero. At time t = 50, the disturbance signal of step input type is introduced in ${T_{jf}}$ having initial value${0^o}F$and final value$4{0^o}F$. Step Disturbance signal is also introduced in ${T_f}$from $6{0^o}F$to final value of $8{0^o}F$at t = 100. The corresponding temperature of the reactor and efforts required to maintain the reactor temperature are illustrated in Figs. 8 and 9, respectively.

**Table 3:** Controller parameters and performance index.

Methods	Controller Settings				Settling time			Y_max_		
		IAE	ISE	ITAE	Y_1_	D_2_	D_1_	Y_1_	D_2_	D_1_
Proposed	${G_{c2}}= - 0.01 - \frac{{0.01}}{s}$ ${G_{c1}}= - 977.3 - \frac{{0.105}}{s} - 47.29s$	47.6	694	2368	3	55	112	101.1	101.19	104.6
CCS	${G_{c2}}= - 0.01 - \frac{{0.01}}{s}$ ${G_{c1}}=131.1+\frac{{4.89}}{s}$	91.9	3093	2839	5	74	131	104.1	94.1	112.03
PCS	${G_{cl}}=\frac{{{\text{-0}}{\text{0.708}}{{\text{s}}^2}{\text{ -1}}{\text{.599s -0}}{\text{0.2168}}}}{{{\text{[s(0}}{\text{.4797s+1)]}}}}$ ${G_{cs}}=\frac{{{\text{-5}}{\text{0.966}}{{\text{s}}^2}{\text{ -1}}{\text{.788s -0}}{\text{0.1074}}}}{{{\text{[s(4}}{\text{.66s+1)]}}}}$	446	7170	1.6e^4^	25	82	147	123.1	93.3	111.8

It is observed from the Fig. 8 that the temperature of the reactor for PCCS reaches the desired temperature in a quick span of time with no overshoot as compared to PCS and CCS. In CCS, there is a slight overshoot whereas, the highest overshoot with sluggish setpoint tracking is observed in case of PCS.

The disturbances in ${T_{jf}}$ are promptly suppressed by the PCCS whereas, long settling time is required by PCS and CCS. Also, disturbances in the feed temperature ${T_f}$ are quickly and smoothly rejected by PCCS. Large overshoot is observed in case of PCS and CCS, but the later one has small settling time in rejecting the load disturbances in the feed temperature${T_f}$. The jacket make-up flowrate required to achieve the desired closed loop performance is large for PCCS as compared to CCS and PCS. The performance measures calculated are shown in Table 3. From the table, it is noted that the least value of *IAE*,* ISE*, *ITAE*, settling time and *Y*_*max*_ are observed for PCCS for both setpoint tracking and load disturbance rejection, thereby, indicating better performance by the proposed method as compared to PCS and CCS.

The *IAE* improvement is also calculated for PCCS over the other structures as

50 $$IAE{\text{ }}improvement=\frac{{IA{E_{of{\text{ }}other{\text{ }}structure}} - IA{E_{of{\text{ P}}CCS}}}}{{IA{E_{of{\text{ P}}CCS}}}} \times 100\%.$$

The *IAE* improvement of PCCS over PCS is very high and is the highest as compared to CCS (93.01%).

**Fig. 10:**
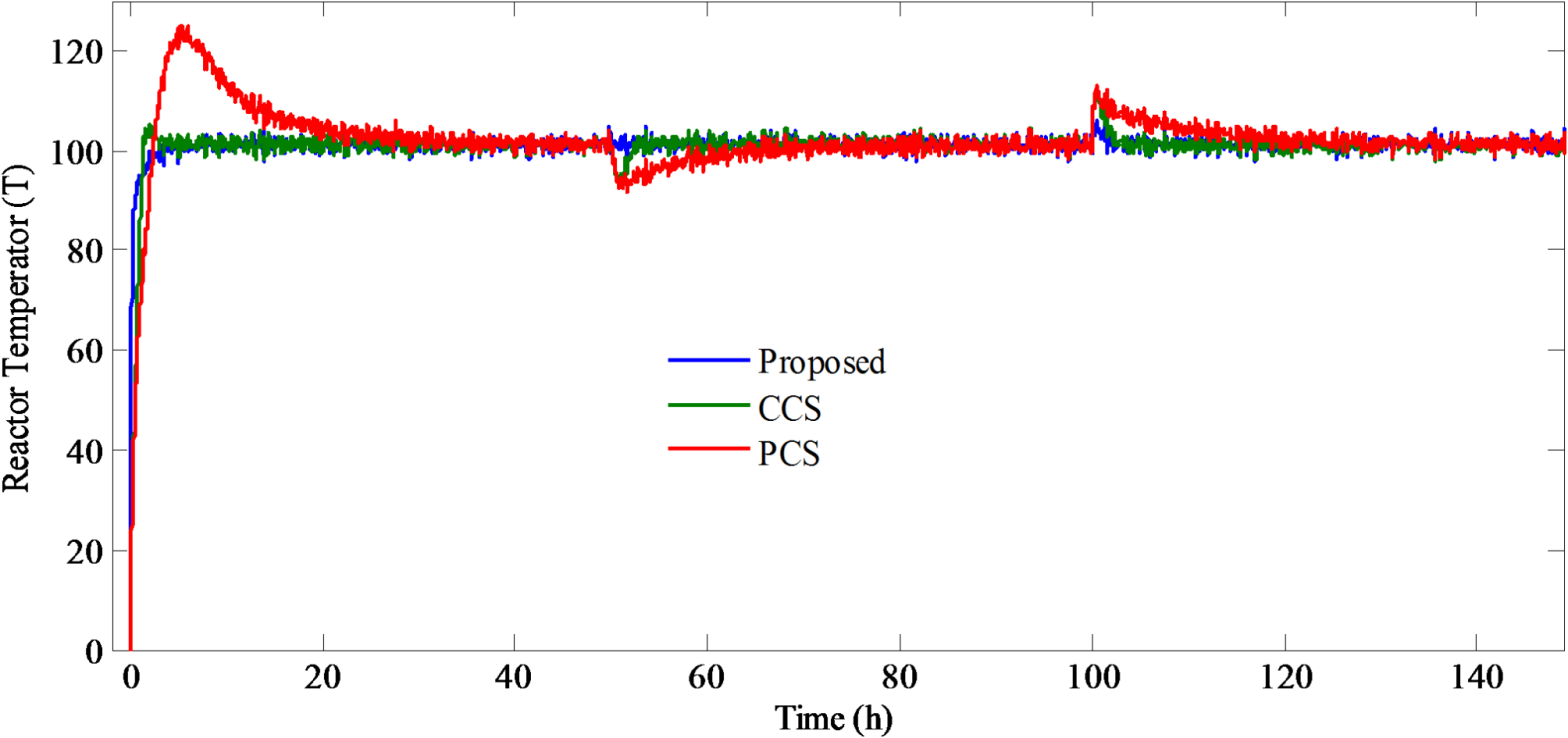
Temperature of the reactor under noisy conditions for PCCS, CCS, and PCS.

Furthermore, to validate the efficiency of the structures in noisy environment, a signal representing noise having power = 0.1, sampling time = 0.1 s and seed = 0 s is applied on the system output. Simulations are carried out and the impact of the white noise on the system output is shown in Fig. 10.

**Fig. 11:**
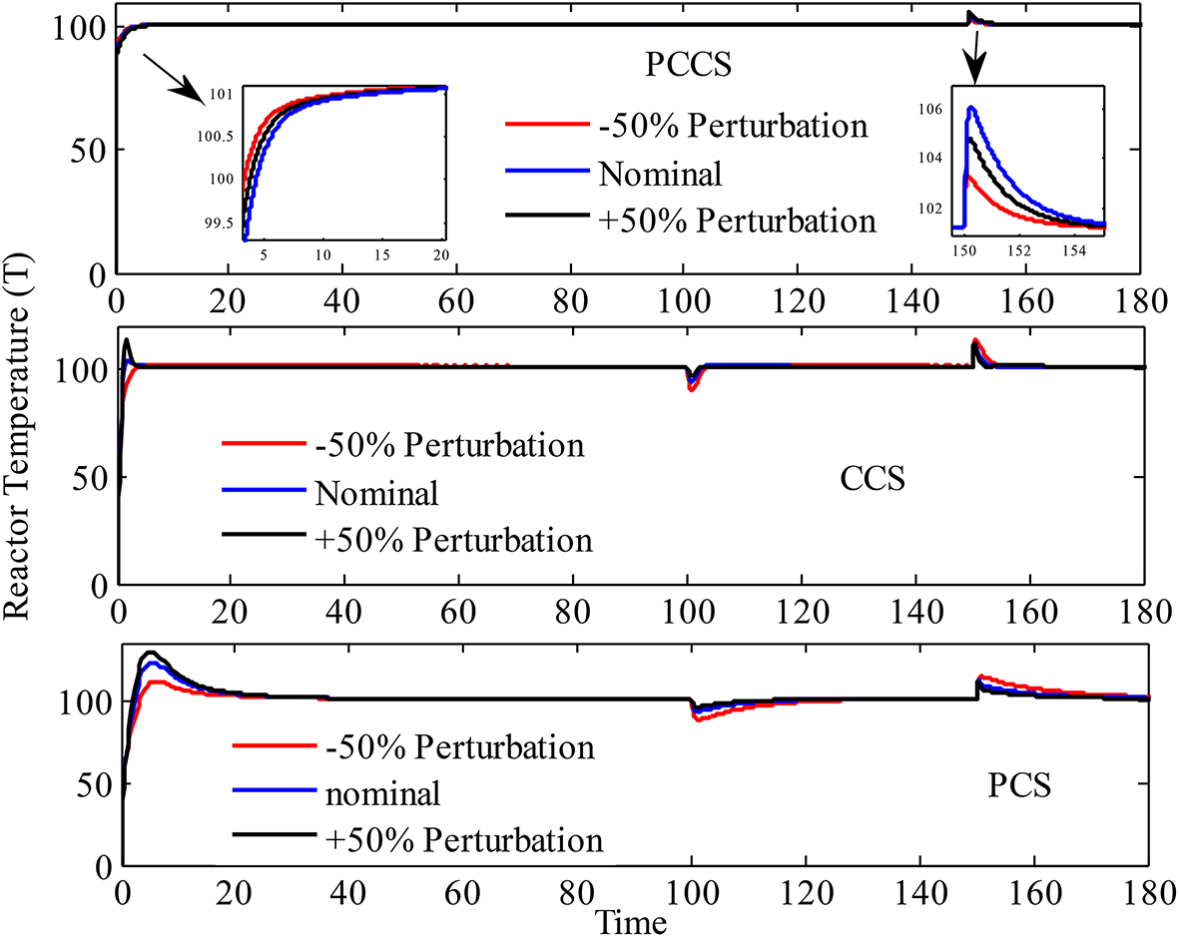
Temperature of the reactor under perturbed conditions for PCCS, CCS, and PCS.

It is observed that all the structures are able to handle the NCSTR in a satisfactory way. To investigate the robustness of the proposed method in PCCS, $\pm 50\%$perturbations are introduced in $\rho$and${\rho _j}$, ${C_p}$and${C_{pj}}$,${A_r}$, *U*, *V* and *V*_*j*_. The resulting response generated in Fig. 11, clearly indicates that PCS and CCS are susceptible to parametric variation as the response gets deviated from the nominal performance whereas, the PCCS indicated strong robustness to the variations in model parameters.

**Fig. 12:**
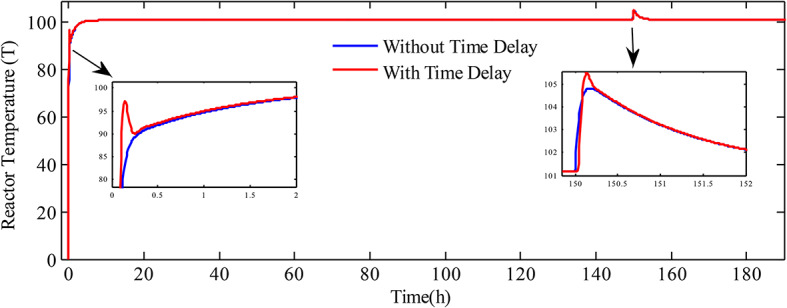
Temperature of the reactor with and without time delay.

In a real-world case, there are inevitably time delays due to non-homogeneous conditions which may change the behavior or performance of NCSTR. Therefore, it is essential to evaluate the performance of the proposed system by considering the time delay. Hence, in this work, a time delay of 0.037 (h) is considered that is taken from the literature^37^. Simulation has been conducted and the response obtained is illustrated in Fig. 12. Figure indicates that the proposed system withstands the effect of time delay and delivers satisfactory performance.

**Fig. 13:**
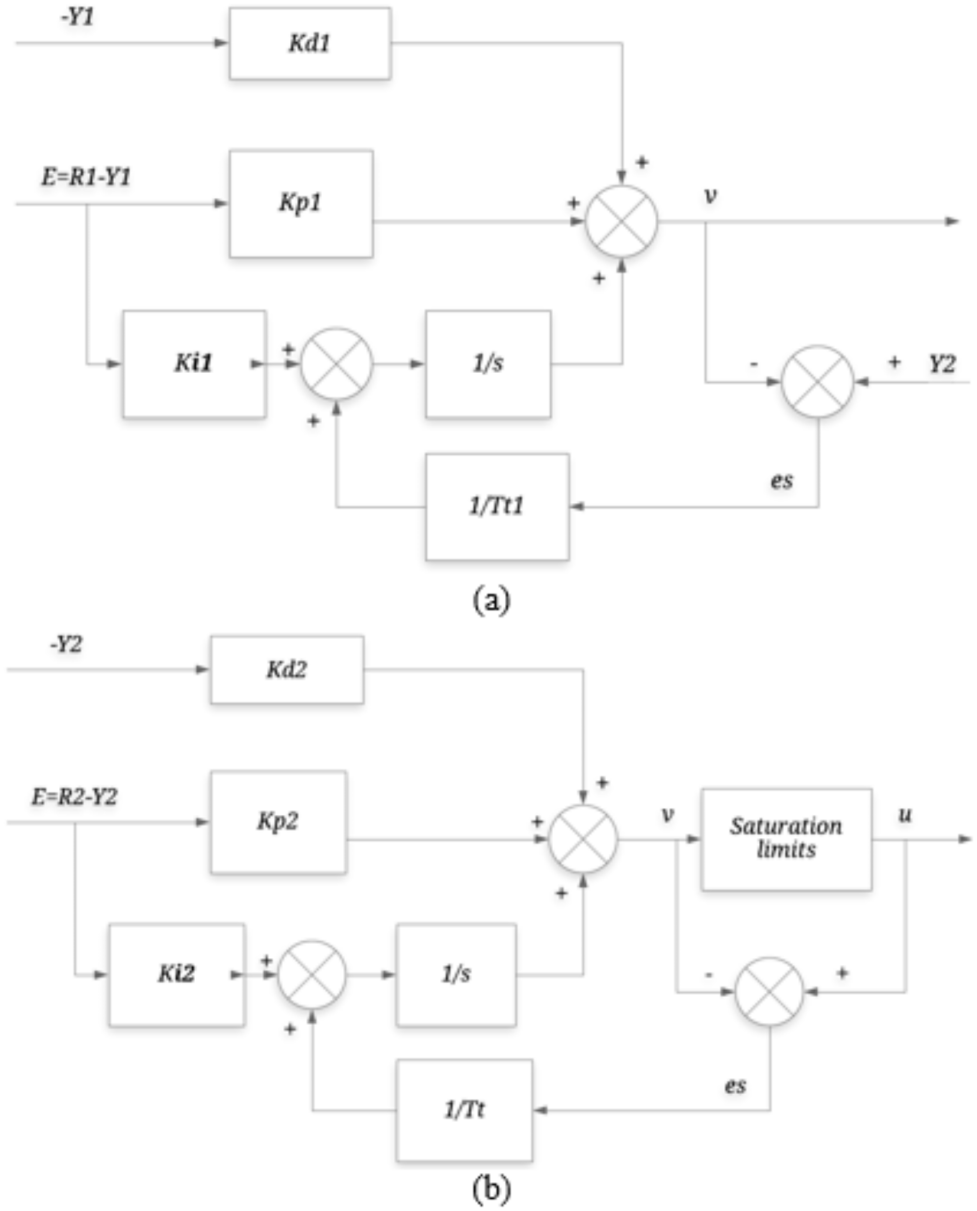
The anti-windup scheme for (**a**) outer loop controller and (**b**) inner loop controller in PCCS.

**Fig. 14:**
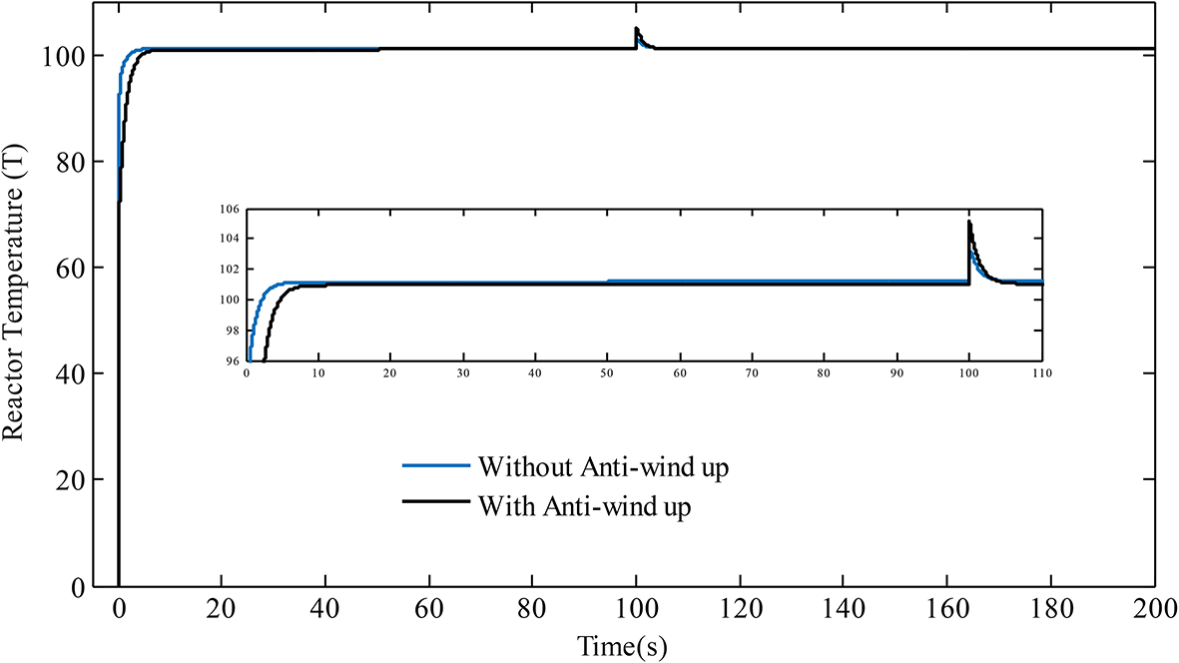
Response with anti-windup and without anti-windup scheme.

The PCCS in this work utilizes a PI controller in the inner loop and a PID controller in the outer loop, so it becomes essential to assess the effect of integral windup linked with integral term that usually saturates the controller efforts. To address this issue, anti-windup approaches are often adopted^38,39^. The proposed approach uses the anti-windup scheme with back calculation methodology as shown in Fig. 13. The limit of the saturation is selected as [− 10 10^3^] and the *T*_*t*_ (tracking time constant) is considered as 0.03*(*K*_*pil*_*/K*_*iil*_) and 0.03*(*K*_*pout*_*/K*_*iout*_) for inner and outer loop, respectively^40^. Simulations are performed by giving a signal of step input having magnitude of $101.{1^o}F$at time equal to zero and step disturbance signal is introduced in ${T_f}$from $6{0^o}F$to final value of $8{0^o}F$at t = 100 to see the effect of anti-windup scheme. The response with and without the anti-windup scheme is generated and is shown in Fig. 14. It can be observed that the performance of the present approach is acceptable with anti-windup scheme.

## Conclusion

A simple controller design approach based on model matching technique in the frequency domain for parallel cascade control structure (PCCS) is suggested to handle the temperature of NCSTR. Enhanced regulatory performance is achieved by selecting the appropriate DCLM_FLD model for the inner loop and performing the model matching in the frequency domain. The outer loop PID controller is designed based on pole placement and model matching technique in the frequency domain for good servo performance. The PCCS with the proposed controller is examined using Kharitonov’s theorem and the PCCS is found to be robustly stable. When comparative analysis is performed, it is observed that the significant improved performance of PCCS has been observed in terms of tracking reactor temperature, promptly rejecting the disturbances in ${T_f}$ (feed temperature) and ${T_{jf}}$ (jacket inlet coolant temperature) as compared CCS and PCS but with large flow of liquid in jacket around the reactor. Under noisy and perturbed situations, the proposed controller in PCCS gave smooth response whereas CCS and PCS have fluctuation. The overall closed loop performance of the PCCS is superior over the CCS and PCS.

## Data Availability

All data generated or analysed during this study are included in this published article.
